# Relationship between lymph nodes examined and survival benefits with postoperative radiotherapy in oral cavity squamous cell carcinoma patients with stage T1-2N1M0

**DOI:** 10.3389/fsurg.2022.928455

**Published:** 2022-09-28

**Authors:** Sufeng Fan, Wenmei Jiang, Zhongyi Fang, Ruiyu Li, Yudong Li, Yanfeng Chen, Quan Zhang

**Affiliations:** ^1^ Department of Oncology, Henan Provincial People’s Hospital, Zhengzhou, China; ^2^ Department of Head and Neck Surgery, Sun Yat-sen University Cancer Center, State Key Laboratory of Oncology in South China, Collaborative Innovation Center for Cancer Medicine, Guangzhou, China

**Keywords:** oral cavity squamous cell carcinoma, postoperative radiotherapy, lymph nodes examined, prognosis, survival analysis

## Abstract

**Background:**

This study aims to explore the relationship between the lymph nodes examined and survival benefits of postoperative radiotherapy in oral cavity squamous cell carcinoma patients with stage T1-2N1M0.

**Methods:**

This study retrieved patients who underwent dissection of the primary site and neck lymph nodes for pT1-2N1M0 oral cavity squamous cell carcinoma without adverse nodal features from the Surveillance, Epidemiology, and End Results database from 2004 to 2015. Propensity score matching analysis was conducted, and the best cutoff value of the lymph nodes examined was determined by X-tile. Cancer-specific survival was the primary outcome. Univariable and multivariable analyses were performed to assess the relation between postoperative radiotherapy and cancer-specific survival, adjusting for other prognostic factors.

**Results:**

A total of 469 patients were finally enrolled according to our exclusion criteria, and then 119 pairs of patients were matched by propensity score matching analysis. The best cutoff value of the lymph nodes examined was determined by X-tile, stratifying patients into lymph nodes examined ≤16 group and lymph nodes examined >16 group. For the whole matched cohort, the choice of postoperative radiotherapy had no correlation with other factors (all *p*’s* *> 0.05), and postoperative radiotherapy made no contribution to a better survival outcome for patients (*p* = 0.289). After stratified by the lymph nodes examined, in the lymph nodes examined ≤16 group, significantly improved CSS was found for those who undertook postoperative radiotherapy compared to those who just received surgery (unadjusted hazard ratio, 0.541; 95% confidence interval, 0.333–0.878; *p* = 0.013).

**Conclusions:**

Our study revealed that pT1-2N1M0 oral cavity squamous cell carcinoma patients were more likely to benefit from postoperative radiotherapy when unsatisfactory neck dissection was conducted, indicating that the number of lymph nodes examined might be a factor when clinicians do therapeutic planning for early-stage oral cavity squamous cell carcinoma patients.

## Background

Head and neck cancers are the seventh most common malignancies in the world, with annually 977,171 new cases and 461,774 deaths, and squamous cell carcinoma (SCC) accounts for approximately 90% of the whole (1). Oral cavity SCC (OCSCC) is the most common malignancy of the head and neck (excluding nonmelanoma skin cancer), with a gradually increasing incidence (2–4). Unlike the development of treatment mode, the 5-year overall survival (OS) rate of OCSCC consistently remained around 50% over the past 30 years (2). For the early stage of OCSCC with pN0, there is a well-supported consensus on the observation after radical surgery if there are no indications for primary site radiotherapy (5–7). According to the updated National Comprehensive Cancer Network (NCCN) guidelines (8), for patients with the pT1-2N1M0 stage after radical dissection of the primary site and neck lymph nodes, if the pathological examination proves a single positive lymph node without risk factors (such as extranodal extension, positive margins, pT3 or pT4 primary, pN2 or pN3 nodal disease, nodal disease in levels IV or V, perineural invasion, vascular invasion, lymphatic invasion), PORT is worth considering. The suggestion of considering might leave space for different advice on PORT for patients with the same pT1-2N1M0 stage.

Until now, there is still no compelling evidence from a prospective randomized study on the decision of whether PORT should be applied to the pT1-2N1M0 OCSCC. Several previous studies supported that PORT contributed to better survival for early-stage OCSCC patients with pT1-2N1 stage (9–11). Based on the growing pool of evidence mainly derived from a retrospective study, the American Society of Clinical Oncology (ASCO) stated more precise suggestions that PORT should be administered to OCSCC patients with pT1-2N1M0 stage when the high-quality neck dissection was not conducted. ASCO defined the high-quality neck dissection as no less than 18 lymph nodes examined (12). For colorectal cancer, less than 12 lymph nodes examined is regarded as a high-risk factor for recurrence in the NCCN guidelines (13). Although several prior studies had demonstrated that patients with over 16–18 lymph nodes examined (LNE) tend to present with a superior OS (14–16), there is still no solid consensus regarding the exact number of lymph nodes examined as used in colorectal cancer to help clinicians to make therapeutic planning for early stage OCSCC patients.

Therefore, to make a contribution to the reference for a better assessment system, our study was designed to explore whether PORT conferred a survival benefit for OCSCC patients with pT1-2N1M0 stage and whether the quality of neck dissection should be a factor to be considered when doctors make decisions on the postoperative treatment.

## Materials and methods

### Data source

The Surveillance, Epidemiology, and End Results (SEER) database was used to extract data on patients diagnosed with OCSCC between 2004 and 2015 for the present study. Sponsored by the National Cancer Institute, the SEER program collects demographic, clinicopathologic, and survival data from 18 population-based cancer registries (SEER-18) in the United States. Since the SEER-18 covers 27.8% of the population in the United States with a typical distribution, it is thought to be representative of the US population as a whole. Information about each patient was retrieved, including age at diagnosis, sex, race, marital status, site of the primary tumor, survival months, vital status, grade, TNM staging (re-evaluated according to the American Joint committee on Cancer (AJCC) TNM staging system, Eighth Edition, 2017), histology, the number of LNE, the number of positive lymph nodes, the receipt of surgery and adjuvant therapy (including radiotherapy and chemotherapy), the sequence of radiotherapy, and causes of death.

### Subjects

**Figure 1:**
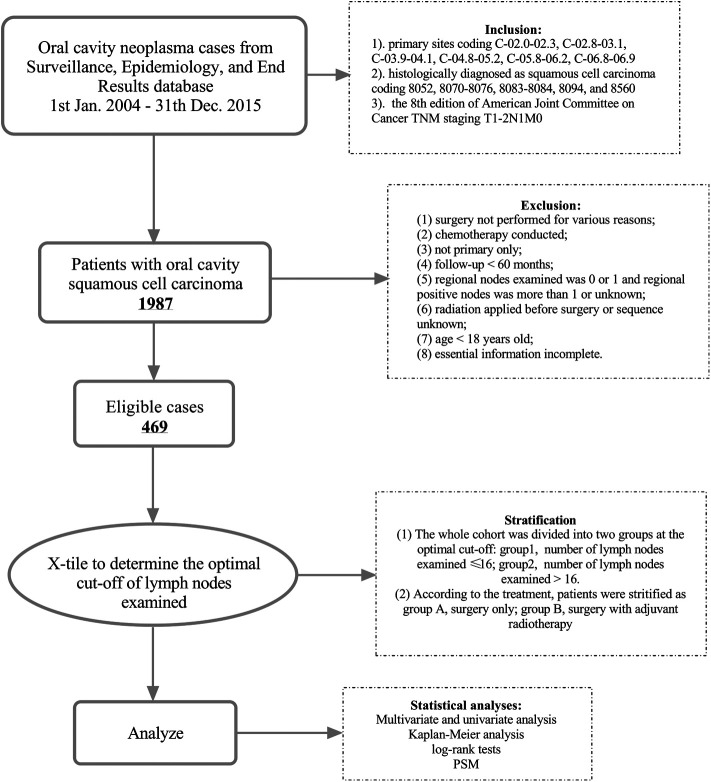
Diagram of the patient screening process in the Surveillance Epidemiology and End Results database.

The study was approved by the Clinical Research Ethics Committee of Sun Yat-sen University Cancer Center (approval number: B2022-201-01), and the informed consent of patients was waived. The process for patient selection is clearly shown in Figure 1. Patients were identified for study according to the International Classification of Diseases for Oncology, Third Edition, topography codes for the oral cavity (tongue C02.0–02.9, gum C03.0–03.9, the floor of mouth C04.0–04.9, palate C05.0–05.9, other parts of mouth C06.0–06.9) and morphologically codes 8052, 8070–8076, 8083–8084, 8094, and 8560 for squamous cell carcinoma. For the purpose of our study, specific patients who started with T1 or T2 stage and N1 stage and without distant metastasis were included. Then, a total of 1,987 cases were retrieved from the SEER database. Ineligible cases were excluded for further analysis according to the following requirements: (1) surgery was not performed for various reasons; (2) chemotherapy was conducted; (3) not one primary only; (4) follow-up <60 months; (5) regional nodes examined was 0 or 1 and regional positive nodes were more than 1 or unknown; (6) radiation was applied before surgery or sequence unknown; (7) age <18 years old; and (8) essential information was incomplete. Finally, 469 patients were enrolled in our study who were diagnosed with pT1-2N1M0 OCSCC and underwent resection of primary carcinoma and neck lymph nodes with or without PORT between 2004 and 2015 in United States. The main outcome of the analysis was CSS, which was defined as the number of months from diagnosis to the date of death due to OCSCC. Those who were still alive or dead of other cancers at the end of the follow-up period were defined as censored.

### Propensity score matching

The number of LNE was analyzed by using the X-tile plot (17, 18) to determine the appropriate cutoff value, and according to the cutoff value, patients were stratified into the LNE ≤ 16 group and LNE > 16 group. To improve the evidence level of the study group, we performed a 1:1 patient pairing (nearest-neighbor matching) by using PSM (19, 20). We used LNE and several other variables (treatment, sex, pT stage, age at diagnosis, race, marital status, and grade) as covariates in this PSM model. Finally, 119 pairs of OCSCC patients were matched after 231 cases were discarded. The differences in the propensity score in each pair were no more than 0.02. Baseline demographic and tumor characteristics stratified by the number of LNE among the matched group are shown in Table 1.

**Table 1:** Associations between lymph nodes examined and clinicopathological characteristics of OCSCC patients (after PSM).

Variables	Total	Lymph nodes examined		*p*
		≤16 (%)	>16 (%)	
Total	238	119 (50)	119 (50)	
Sex				0.296*
Male	134 (56.3)	71 (59.7)	63 (52.9)	
Female	104 (43.7)	48 (40.3)	56 (47.1)	
Adjuvant radiotherapy				0.598^a^
No	98 (41.2)	51 (42.9)	47 (39.5)	
Yes	140 (58.8)	68 (57.1)	72 (60.5)	
Age (years)				0.108^a^
≤60	95 (39.9)	55 (46.2)	40 (33.6)	
60–80	126 (52.9)	55 (46.2)	71 (59.7)	
>80	17 (7.2)	9 (7.6)	8 (6.7)	
Grade				0.358^a^
Well	24 (10.1)	11 (9.2)	13 (10.9)	
Moderate	163 (68.5)	78 (65.5)	85 (71.4)	
Poor	51 (21.4)	30 (25.3)	21 (17.6)	
Subsite				0.307^a^
Tongue	153 (64.3)	83 (69.7)	70 (58.8)	
Gum	16 (6.7)	9 (7.6)	7 (5.9)	
Floor of mouth	31 (13.0)	13 (10.9)	18 (15.1)	
Palate	5 (2.1)	2 (1.7)	3 (2.5)	
Others	33 (13.9)	12 (10.1)	21 (17.7)	
pT stage				0.052^a^
T1	125 (52.5)	55 (46.2)	70 (58.8)	
T2	113 (47.5)	64 (53.8)	49 (41.2)	
Race				0.749^a^
White	202 (84.9)	103 (86.6)	99 (83.2)	
Black	12 (5.0)	5 (4.2)	7 (5.9)	
Other	24 (10.1)	11 (9.2)	13 (10.9)	
Marital status				0.677^b^
Married	147 (61.8)	73 (61.3)	74 (62.1)	
Unmarried	85 (35.7)	44 (37.0)	41 (34.5)	
Unknown	6 (2.5)	2 (1.7)	4 (3.4)	

PSM, propensity score matching; LNE, lymph nodes examined.

^a^ *χ*^2^ test.

^b^ Fisher’s exact test.

### Statistical analysis

We used frequencies and proportions for categorical variables to describe the patient characteristics and compared the differences between groups using the *χ*^2^ test and Fisher’s exact test. The Kaplan–Meier method and the log-rank test were performed to evaluate the role of treatment in the survival of OCSCC patients. We also performed univariate and multivariate Cox regression analysis was used to identify the independent risk factors of OCSCC patients. Hazard ratios (HRs) with 95% confidence intervals (95% CIs) were calculated by univariable and multivariable Cox proportional hazard regression analyses. All statistical tests were two-sided, with statistical significance evaluated at the 0.05 level. All calculations were performed using SPSS Statistics 25.0 software (IBM SPSS, Inc., Chicago, IL, USA) and X-tile software version 3.6.1 (http://tissuearray.org).

## Results

### Patients demographics

From January 2004 to December 2015, there were a total of 1,987 patients with pT1-2N1M0 OCSCC, and only 469 patients were enrolled in our study according to the criteria before PSM with a median age of 60 years old (range, 20–91). The median survival time was 40 months (range, 2–155). For the whole cohort, the tongue was the most common primary site, and the majority was moderately differentiated squamous cell carcinoma. Males make up 57.4% of the total, and the males were slightly more likely to accept PORT compared with the female group but without statistical significance (59.5% vs. 53.5%, *p* = 0.196). The detailed characteristics of patients before PSM are listed in Supplementary Table S1. According to the *χ*^2^ test and Fisher’s exact test based on the analysis of sex, LNE, age, grade, subsite, pT stage, race, marital status, and treatment, whether OCSCC patients chose to take PORT after radical surgery had no statistical relationship with these factors mentioned above, except marital status (all *p*'s > 0.05, Supplementary Table S1).

After PSM, 119 pairs of patients were matched (according to the number of lymph nodes examined), and the clinicopathological characteristics are listed in Table 1. In the matched cohort, the median age was 63 years old (range, 21–90), and the median survival time was 32.5 months (range, 2–155). The majority were male (56.3%), married (61.8%), and white (84.9%). In the matched cohort, 153 patients (64.3%) died and 115 (71.2%) of the death were specific to OCSCC. We found that whether OCSCC patients chose to take PORT after radical surgery had no statistical relationship with other factors (all *p*'s > 0.05, Table 1).

### Relationship between PORT and CSS of LNE stratified patients

**Figure 2:**
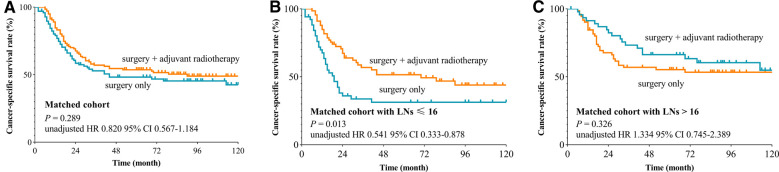
Cancer-specific survival curve for oral cavity squamous cancer patients with stage T1-2N1M0 according to the treatment approaches in the matched cohort of Surveillance Epidemiology and End Results database. (**A**) Whole cohort, (**B**) patients with lymph nodes examined ≤16, and (**C**) patients with lymph nodes examined >16).

**Figure 3:**
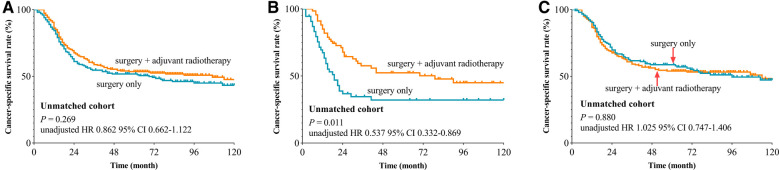
Cancer-specific survival curve for oral cavity squamous cancer patients with stage T1-2N1M0 according to the treatment approaches in the unmatched cohort of Surveillance Epidemiology and End Results database. (**A**) Whole cohort, (**B**) patients with lymph nodes examined ≤16, and (**C**) patients with lymph nodes examined >16).

We used the X-tile plot to determine the best cutoff value on the number of LNE, and it turned out to be 16. Then, the whole group was divided into the LNE ≤ 16 group and LNE > 16 group. According to the results of Kaplan–Meier analysis, the CSS revealed no difference between the surgery-only group and PORT group both after (*p* = 0.289, Figure 2A) and before (*p* = 0.269, Figure 3A) PSM. When stratified by LNE, for the LNE ≤ 16 group, patients who underwent PORT had superior survival compared with that those who took surgery only both after (*p* = 0.013, Figure 2B) and before (*p* = 0.011, Figure 3B) PSM. In contrast, the results revealed no significance in the LNE > 16 group both after (*p* = 0.326, Figure 2C) and before (*p* = 0.880, Figure 3C) PSM.

In the matched group, we performed a univariate analysis based on sex, age, grade, LNE, treatment, subsite, T stage, race, and marital status and found that for the whole group, LNE (*p* = 0.007), subsite (*p* = 0.046), and marital status (*p* = 0.001, Table 2) were statistically associated with survival, while for the LNE ≤ 16 group, sex (*p* = 0.005), treatment (*p* = 0.013), and marital status (*p* = 0.001) were statistically significant in the univariate COX regression analysis, and then these factors were included into multivariate COX regression analysis, indicating that sex, treatment, and marital status were independent risk factors for OCSCC (all *p* < 0.05, Table 3). Females, compared with males, had an HR of 1.814 (95% CI, 1.107, 2.974; *p* = 0.018). Compared with patients who underwent surgery only, patients with PORT had an HR of 0.557 (95% CI, 0.340, 0.912; *p* = 0.020), and the unmarried group had an HR of 2.276 (95% CI, 1.371, 3.777; *p* = 0.001) compared with the married group.

**Table 2:** Univariable and multivariable analyses of CSS by the Cox regression model before and after PSM.

Variables	Univariable analysis		Multivariable analysis	
	HR (95% CI)	*p-*value	HR (95% CI)	*p-*value
Before PSM				
Sex				
Male	1 (ref)			
Female	1.231 (0.945, 1.602)	0.124		
Age (years)				
≤60	1 (ref)			
60–80	1.079 (0.821, 1.418)	0.587		
>80	1.578 (0.921, 2.705)	0.097		
Grade				
Well	1 (ref)			
Moderate	1.468 (0.850, 2.535)	0.168		
Poor	1.730 (0.955, 3.134)	0.071		
Lymph nodes examined				
≤16	1 (ref)		1 (ref)	
>16	0.686 (0.515, 0.914)	**0**.**010**	0.653 (0.488, 0.872)	**0**.**004**
Treatment				
Surgery only	1 (ref)			
Radiation after surgery	1.160 (0.892, 1.510)	0.269		
Subsite				
Tongue	1 (ref)			
Gum	1.313 (0.781, 2.208)	0.304		
Floor of mouth	1.251 (0.872, 1.797)	0.224		
Palate	1.553 (0.815, 2.957)	0.181		
Other	1.098 (0.734, 1.642)	0.650		
pT stage				
T1	1 (ref)			
T2	1.243 (0.955, 1.618)	0.106		
Race				
White	1 (ref)			
Black	1.435 (0.834, 2.469)	0.192		
Other	1.290 (0.805, 2.067)	0.290		
Marital status				
Married	1 (ref)		1 (ref)	
Unmarried	1.394 (1.068, 1.820)	**0**.**015**	1.474 (1.125, 1.931)	**0**.**005**
Unknown	0.488 (0.180, 1.322)	**0.158**	0.520 (0.192, 1.408)	**0.198**
After PSM				
Sex				
Male	1 (ref)			
Female	1.385 (0.960, 1.998)	0.081		
Age (years)				
≤60	1 (ref)			
60–80	1.082 (0.739, 1.584)	0.686		
>80	1.264 (0.598, 2.674)	0.540		
Grade				
Well	1 (ref)			
Moderate	1.693 (0.782, 3.664)	0.181		
Poor	1.962 (0.852, 4.521)	0.113		
Lymph nodes examined				
≤16	1 (ref)		1 (ref)	
>16	1.668 (1.151, 2.415)	**0**.**007**	0.582 (0.397, 0.855)	**0**.**006**
Treatment				
Surgery only	1 (ref)		1 (ref)	
PORT	0.820 (0.567, 1.184)	0.289	0.797 (0.548, 1.159)	0.234
Subsite				
Tongue	1 (ref)		1 (ref)	
Gum	1.520 (0.760, 3.043)	0.237	1.510 (0.749, 3.044)	0.250
Floor of mouth	1.693 (1.008, 2.843)	**0**.**046**	1.535 (0.903, 2.609)	0.113
Palate	0.815 (0.200, 3.325)	0.776	0.889 (0.217, 3.636)	0.870
Other	0.979 (0.552, 1.736)	0.942	1.040 (0.579, 1.866)	0.896
pT stage				
T1	1 (ref)		1 (ref)	
T2	1.312 (0.910, 1.893)	0.146	1.217 (0.837, 1.769)	0.303
Race				
White	1 (ref)			
Black	0.932 (0.408, 2.126)	0.867		
Other	1.000 (0.549, 1.822)	1.000		
Marital status				
Married	1 (ref)		1 (ref)	
Unmarried	1.930 (1.329, 2.803)	**0**.**001**	1.900 (1.292, 2.795)	**0**.**001**
Unknown	0.763 (0.185, 3.097)	0.741	0.786 (0.189, 3.269)	0.741

PSM, propensity score matching; HR, hazard ratio; CI, confidence interval; CSS, cancer-specific survival; PORT, postoperative radiotherapy.

* The significance of boldface values is *P*-value <0.05.

**Table 3:** Univariable and multivariable analyses of CSS by the Cox regression model (after PSM and lymph nodes examined ≤16).

Variables	Univariable analysis		Multivariable analysis	
	HR (95% CI)	*p-*value	HR (95% CI)	*p-*value
Sex				
Male	1 (ref)		1 (ref)	
Female	1.994 (1.227, 3.241)	**0**.**005**	1.814 (1.107, 2.974)	**0**.**018**
Age (years)				
≤60	1 (ref)			
60–80	1.502 (0.907, 2.487)	0.114		
>80	1.119 (0.434, 2.887)	0.815		** **
Grade				
Well	1 (ref)			
Moderate	2.494 (0.776, 8.010)	0.125		
Poor	2.368 (0.685, 8.184)	0.173		
Treatment				
Surgery only	1 (ref)		1 (ref)	
PORT	0.541 (0.333, 0.878)	**0**.**013**	0.557 (0.340, 0.912)	**0**.**020**
Subsite				
Tongue	1 (ref)			
Gum	0.747 (0.269, 2.077)	0.576		
Floor of mouth	1.361 (0.641, 2.891)	0.422		
Palate	0.996 (0.137, 7.233)	0.997		
Other	1.464 (0.661, 3.247)	0.348		
pT stage				
T1	1 (ref)			
T2	1.177 (0.725, 1.910)	0.510		
Race				
White	1 (ref)			
Black	1.042 (0.326, 3.333)	0.944		
Other	1.430 (0.649, 3.149)	0.374		
Marital status				
Married	1 (ref)		1 (ref)	
Unmarried	2.383 (1.453, 3.909)	**0**.**001**	2.276 (1.371, 3.777)	**0**.**001**
Unknown	0.804 (0.110, 5.871)	0.830	0.935 (0.126, 6.946)	0.947

PSM, propensity score matching; HR, hazard ratio; CI, confidence interval; CSS, cancer-specific survival; PORT, postoperative radiotherapy.

* The significance of boldface values is *P*-value <0.05.

## Discussion

Despite the increasing incidence of OCSCC in the United States (3, 4, 21), limited evidence was provided by the pool of prospective studies on the inconsistent consensus regarding whether PORT ought to be applied to OCSCC patients with pT1-2N1M0 stage for the improved survival benefit. According to the NCCN guidelines, there is no exact suggestion on whether PORT should be applied to patients who had only one pathologically positive lymph node without adverse risk features (such as extranodal extension, positive margins, pT3 or pT4 primary, pN2 or pN3 nodal disease, nodal disease in levels IV or V, perineural invasion, vascular invasion, and lymphatic invasion) (8). Also, studies regarding the impact of PORT on the survival of patients with OCSCC were variable. Shrime et al. retrospectively studied OCSCC patients based on the analysis of the SEER database from 1983 to 2004. It is reported that PORT induced an improved OS and CSS among OCSCC patients with T2N1 stage, while no difference was found for the T1N1 group (10). In another study, Chen et al. found that PORT conferred obviously elevated OS for OCSCC patients with T1-2N1 stage, especially for those younger than 70 years or those with T2 disease, by reviewing the National Cancer Database from 2004 to 2013 (11). ASCO recommended that pT1-2N1M0 patients without adverse risk factors did not need to undergo PORT if adequate neck dissection was conducted. Nevertheless, no direct research focused on the association between PORT and the survival benefit for OCSCC patients with pT1-2N1M0 stage, especially when they were stratified by the number of LNE.

Thus, our study retrieved the demographic and clinical information of OCSCC patients from the SEER database (2004–2015) and performed a 1:1 patient pairing by PSM to reduce bias. The analysis of these highly matched 119 pairs of patients revealed that the pT1-2N1M0 OCSCC patients without adverse nodal features had no statistically different CSS, regardless of whether they took PORT. We also found that whether patients took PORT or not had no statistical significance with other factors. Also, after the stratification of LNE, in the LNE ≤ 16 group, PORT was the independent factor associated with statistically significant superior CSS, while the difference was absent in the LNE > 16 group.

It is noticeable that the study of Torrecillas et al. analyzed the patients from SEER in nearly the same period (year ranging from 2004 to 2013) as our study, but the results were totally different. They indicated that treatment with adjuvant radiation therapy was significantly associated with improved 5-year CSS and 5-year OS for patients with T1-2N1 tumors (9). The reason responsible for the difference might lie in that we excluded the patients whose lymph nodes examined were 0 or 1, trying to avoid enrolling patients who might just take a biopsy instead of therapeutic neck dissection. Therefore, stage migration could be avoided to some extent.

Although the benefit of neck dissection in patients with early-stage oral cavity cancer has been controversial over the decades (22), a prospective, randomized clinical trial has shed light on this controversy (23). D'Cruz et al. compared patients of cT1-2N0M0 oral cancers who underwent elective neck dissection along with glossectomy with those who underwent therapeutic neck dissection only when regional metastasis happened. The results indicated that those who received upfront elective neck dissection had significantly improved OS and CSS compared with those who took therapeutic dissection afterward. The enrollment of those who only took the biopsy of neck lymph nodes might turn the results in a different direction as the absence of neck dissection may mix upper N stage with pN1, and the former group was demonstrated to benefit certainly from PORT (24, 25). Based on this evidence, we excluded the patients without neck dissection, as we mentioned above. Furthermore, our group had set rather tough exclusions on the database, like the exclusion of those with chemotherapy conducted and a follow-up of <60 months, trying to reduce the disturbance from other confounding factors by PSM.

We found that whether patients took PORT or not had no statistical significance with other factors (all *p*'s < 0.05). Moreover, after the stratification of LNE, PORT was the independent factor associated with statistically significant superior CSS in the LNE ≤ 16 group, while the difference was absent in the LNE > 16 group. Unlike the well-established metrics of LNE for colorectal cancer (13), there was not an exact threshold of LNE as a predictor of survival for patients with OCSCC. Several prior studies have demonstrated that patients with over 16–18 LNE tend to present with a superior OS (14–16). In addition, Kuo et al. reported that survival of the whole group improved with higher lymph node yields (14). In this present study, the best cutoff value of LNE was identified as 16 using X-tile. Several reasons may be the potential explanations for the superior survival for those who underwent PORT compared with those who underwent surgery only in the LNE ≤ 16 group. Unsatisfactory neck dissection might reduce the diagnostic benefit by removing insufficient lymph nodes, detecting a low incidence of extracapsular spread, and facilitating an inaccurate N staging, which subsequently might interfere with the decisions on adjuvant treatment (23). Several studies had stated that extensive neck dissection could certainly reduce regional recurrence, which might be compensated by PORT among patients who underwent unsatisfactory neck dissection or with advanced N stage (25–27). Margin status has been reported to be a strong independent predictor of local recurrence and OS by previous studies (28). Perineural invasion and lymphovascular invasion were reported to correlate with inferior 5-year outcomes for early-stage patients, and they are also strong predictors of locoregional failure (29–33). However, these important factors were not recorded in the SEER database, which might reduce the confidence of our study. The unmarried status was found to correlate with decreased CSS, including windowed, single, separated, and divorced. It has been well discussed that marriage acts as a significant protective factor for various carcinomas in an extensive extent of literature, as married patients are more likely to undertake aggressive treatment and enjoy more comprehensive care both physically and psychologically, inducing less likelihood to die from carcinomas (34, 35).

Limitations are inherent to this retrospective study based on the SEER database. The most obvious limitation of the database is that there is no specific record regarding whether patients took unilateral or bilateral neck dissections. Therefore, we could not exclude the patients whose positive lymph node came from the contralateral neck, and they were supposed to be stated as pN2c. Another limitation is that the information about some detailed pathological features is absent, as margin status, lymphovascular invasion, and perineural invasion were not available in SEER, which was demonstrated to make a contribution to the prognosis. Therefore, given the natural defects in the retrospective study, more prospective and randomized research is needed to validate our findings further.

## Conclusions

Our study revealed that pT1-2N1M0 OCSCC patients were more likely to benefit from PORT when unsatisfactory neck dissection was conducted, indicating the number of lymph nodes examined might be a factor when clinicians do therapeutic planning for early-stage OCSCC patients.

## Data Availability

The raw data supporting the conclusions of this article will be made available by the authors, without undue reservation.

## References

[1] Sung H Ferlay J Siegel RL Laversanne M Soerjomataram I Jemal A Global cancer statistics 2020: GLOBOCAN estimates of incidence and mortality worldwide for 36 cancers in 185 countries. CA Cancer J Clin. (2021) 71(3):209–49. 10.3322/caac.21660 33538338

[2] Carvalho AL Nishimoto IN Califano JA Kowalski LP. Trends in incidence and prognosis for head and neck cancer in the United States: a site-specific analysis of the SEER database. Int J Cancer. (2005) 114(5):806–16. 10.1002/ijc.20740 15609302

[3] Patel SC Carpenter WR Tyree S Couch ME Weissler M Hackman T Increasing incidence of oral tongue squamous cell carcinoma in young white women, age 18–44 years. J Clin Oncol. (2011) 29(11):1488–94. 10.1200/JCO.2010.31.7883 21383286

[4] Shiboski CH Schmidt BL Jordan RCK. Tongue and tonsil carcinoma: increasing trends in the U.S. population ages 20–44 years. Cancer. (2005) 103(9):1843–9. 10.1002/cncr.20998 15772957

[5] Brown JS Shaw RJ Bekiroglu F Rogers SN. Systematic review of the current evidence in the use of postoperative radiotherapy for oral squamous cell carcinoma. Br J Oral Maxillofac Surg. (2012) 50(6):481–9. 10.1016/j.bjoms.2011.08.014 22196145

[6] Chatzistefanou I Lubek J Markou K Ord RA. The role of neck dissection and postoperative adjuvant radiotherapy in cN0 patients with PNI-positive squamous cell carcinoma of the oral cavity. Oral Oncol. (2014) 50(8):753–8. 10.1016/j.oraloncology.2014.05.005 24909939

[7] Liao C-T Lin C-Y Fan K-H Identification of a high-risk subgroup of patients with resected pT3 oral cavity cancer in need of postoperative adjuvant therapy. Ann Surg Oncol. (2011) 18(9):2569–78. 10.1245/s10434-011-1616-4 21360248

[8] Ajani JA D’Amico TA Bentrem DJ Chao J Cooke D Corvera C Gastric cancer, version 2.2022, NCCN clinical practice guidelines in oncology. J Natl Compr Canc Netw. (2022) 20(2):167–92. 10.6004/jnccn.2022.0008 35130500

[9] Torrecillas V Shepherd HM Francis S Buchmann LO Monroe MM Lloyd S Adjuvant radiation for T1-2N1 oral cavity cancer survival outcomes and utilization treatment trends: analysis of the SEER database. Oral Oncol. (2018) 85:1–7. 10.1016/j.oraloncology.2018.07.019 30220313

[10] Shrime MG Gullane PJ Dawson L Kim J Gilbert RW Irish JC The impact of adjuvant radiotherapy on survival in T1-2N1 squamous cell carcinoma of the oral cavity. Arch Otolaryngol. (2010) 136(3):225–8. 10.1001/archoto.2010.22 20231637

[11] Chen MM Harris JP Hara W Sirjani D Divi V. Association of postoperative radiotherapy with survival in patients with N1 oral cavity and oropharyngeal squamous cell carcinoma. JAMA Otolaryngol Head Neck Surg. (2016) 142(12):1224–30. 10.1001/jamaoto.2016.3519 27832255

[12] Koyfman SA Ismaila N Crook D D'Cruz A Rodriguez CP Sher DJ Management of the neck in squamous cell carcinoma of the oral cavity and oropharynx: ASCO clinical practice guideline. J Clin Oncol. (2019) 37(20):1753–74. 10.1200/JCO.18.01921 30811281 PMC7098829

[13] Benson AB Venook AP Al-Hawary MM Cederquist L Chen YJ Ciombor KK NCCN guidelines insights: colon cancer, version 2.2018. J Natl Compr Canc Netw. (2018) 16(4):359–69. 10.6004/jnccn.2018.0021 29632055 PMC10184502

[14] Kuo P Mehra S Sosa JA Roman SA Husain ZA Burtness BA Proposing prognostic thresholds for lymph node yield in clinically lymph node-negative and lymph node-positive cancers of the oral cavity. Cancer. (2016) 122(23):3624–31. 10.1002/cncr.30227 27479645

[15] Huang T Li K Choi W. Lymph node ratio as prognostic variable in oral squamous cell carcinomas: systematic review and meta-analysis. Oral Oncol. (2019) 89:133–43. 10.1016/j.oraloncology.2018.12.032 30732951

[16] Divi V Harris J Harari PM Cooper JS McHugh J Bell D Establishing quality indicators for neck dissection: correlating the number of lymph nodes with oncologic outcomes (NRG oncology RTOG 9501 and RTOG 0234). Cancer. (2016) 122(22):3464–71. 10.1002/cncr.30204 27419843 PMC5237619

[17] Wu LL Liu X Jiang WM Huang W Lin P Long H Stratification of patients with stage IB NSCLC based on the 8th edition of the American Joint Committee on Cancer (AJCC) staging manual. Front Oncol. (2020) 10:571. 10.3389/fonc.2020.00571 32373536 PMC7186345

[18] Camp R Dolled-Filhart M Rimm D. X-tile: a new bio-informatics tool for biomarker assessment and outcome-based cut-point optimization. Clin Cancer Res. (2004) 10(21):7252–9. 10.1158/1078-0432.CCR-04-0713 15534099

[19] Benedetto U Head SJ Angelini GD Blackstone EH. Statistical primer: propensity score matching and its alternatives. Eur J Cardiothorac Surg. (2018) 53(6):1112–7. 10.1093/ejcts/ezy167 29684154

[20] Huang W Wu L Liu X Long H Rong T Ma G. Preoperative serum C-reactive protein levels and postoperative survival in patients with esophageal squamous cell carcinoma: a propensity score matching analysis. J Cardiothorac Surg. (2019) 14(1):167. 10.1186/s13019-019-0981-0 31533862 PMC6751901

[21] Shiboski CH Shiboski SC Silverman S Jr. Trends in oral cancer rates in the United States, 1973–1996. Community Dent Oral Epidemiol. (2000) 28(4):249–56. 10.1034/j.1600-0528.2000.280402.x 10901403

[22] Bulsara VM Worthington HV Glenny AM Clarkson JE Conway DI Macluskey M. Interventions for the treatment of oral and oropharyngeal cancers: surgical treatment. Cochrane Database Syst Rev. (2018) 12(12):CD006205. 10.1002/14651858 30582609 PMC6517307

[23] D'Cruz AK Vaish R Kapre N Dandekar M Gupta S Hawaldar R Elective versus therapeutic neck dissection in node-negative oral cancer. N Engl J Med. (2015) 373(6):521–9. 10.1056/NEJMoa1506007 26027881

[24] Cooper JS Pajak TF Forastiere A Jacobs J Fu KK Ang KK Precisely defining high-risk operable head and neck tumors based on RTOG #85-03 and #88-24: targets for postoperative radiochemotherapy? Head Neck. (1998) 20(7):588–94. 10.1002/(SICI)1097-0347(199810)20:7<588::AID-HED2>3.0.CO;2-F 9744457

[25] Langendijk JA Slotman BJ van der Waal I Doornaert P Berkof J Leemans CR. Risk-group definition by recursive partitioning analysis of patients with squamous cell head and neck carcinoma treated with surgery and postoperative radiotherapy. Cancer. (2005) 104(7):1408–17. 10.1002/cncr.21340 16130134

[26] Ambrosch P Kron M Pradier O Steiner W. Efficacy of selective neck dissection: a review of 503 cases of elective and therapeutic treatment of the neck in squamous cell carcinoma of the upper aerodigestive tract. Otolaryngol Head Neck Surg. (2001) 124(2):180–7. 10.1067/mhn.2001.111598 11226954

[27] Liao CT Hsueh C Lee LY Lin CY Fan KH Wang HM Neck dissection field and lymph node density predict prognosis in patients with oral cavity cancer and pathological node metastases treated with adjuvant therapy. Oral Oncol. (2012) 48(4):329–36. 10.1016/j.oraloncology.2011.10.017 22104249

[28] Buchakjian MR Ginader T Tasche KK Pagedar NA Smith BJ Sperry SM. Independent predictors of prognosis based on oral cavity squamous cell carcinoma surgical margins. Otolaryngol Head Neck Surg. (2018) 159(4):675–82. 10.1177/0194599818773070 29737907 PMC6341475

[29] Lee LY Lin CY Cheng NM Tsai CY Hsueh C Fan KH Poor tumor differentiation is an independent adverse prognostic variable in patients with locally advanced oral cavity cancer–comparison with pathological risk factors according to the NCCN guidelines. Cancer Med. (2021) 10(19):6627–41. 10.1002/cam4.4195 34533269 PMC8495291

[30] Tai SK Li WY Chu PY Chang SY Tsai TL Wang YF Risks and clinical implications of perineural invasion in T1-2 oral tongue squamous cell carcinoma. Head Neck. (2012) 34(7):994–1001. 10.1002/hed.21846 21953773

[31] Tai SK Li WY Yang MH Chu PY Wang YF Chang PM. Perineural invasion as a major determinant for the aggressiveness associated with increased tumor thickness in t1-2 oral tongue and buccal squamous cell carcinoma. Ann Surg Oncol. (2013) 20(11):3568–74. 10.1245/s10434-013-3068-5 23838906

[32] Lanzer M Gander T Kruse A Luebbers HT Reinisch S. Influence of histopathologic factors on pattern of metastasis in squamous cell carcinoma of the head and neck. Laryngoscope. (2014) 124(5):E160–6. 10.1002/lary.24458 24254388

[33] Cheng NM Kang CJ Tsai CY Lee LY Lin CY Hsueh C Improved prognostic stratification of patients with pN3b oral cavity cancer based on maximum standardized uptake value of metastatic nodes, lymph node ratio, and level of cervical nodal metastases. Oral Oncol. (2021) 123:105593. 10.1016/j.oraloncology.2021.105593 34768211

[34] Aizer AA Chen MH McCarthy EP Mendu ML Koo S Wilhite TJ Marital status and survival in patients with cancer. J Clin Oncol. (2013) 31(31):3869–76. 10.1200/JCO.2013.49.6489 24062405 PMC4878087

[35] Rendall MS Weden MM Favreault MM Waldron H. The protective effect of marriage for survival: a review and update. Demography. (2011) 48(2):481–506. 10.1007/s13524-011-0032-5 21526396

