# Supplementary material for: Relationship between lymph nodes examined and survival benefits with postoperative radiotherapy in oral cavity squamous cell carcinoma patients with stage T1-2N1M0
Source: Front Surg. 2022 Sep 28;9:928455. doi: 10.3389/fsurg.2022.928455 (PMC9554262; doi:10.3389/fsurg.2022.928455)
Supplement: Supplementary file 1 [file Table1.docx]

**Table S1. Associations between lymph nodes examined or postoperative radiotherapy and clinicopathological characteristics of OCSCC patients before PSM**.

| **Variables** | **Total** | **LNE** | | ***P*** | **PORT** | | ***P*** |
| --- | --- | --- | --- | --- | --- | --- | --- |
|  |  | **≤ 16 (%)** | **> 16 (%)** |  | **Yes (%)** | **No (%)** |  |
| **Total** | 469 | 123 (26.2) | 346 (73.8) |  | 267 (56.9) | 202 (43.1) |  |
| **LNE** |  |  |  |  |  |  | 0.828 |
| ≤ 16 | 123 |  |  |  | 69 (25.8) | 54 (26.7) |  |
| > 16 | 346 |  |  |  | 198 (74.2) | 148 (73.3) |  |
| **PORT** |  |  |  | 0.828 |  |  |  |
| Yes | 267 | 69 (56.1) | 198 (57.2) |  |  |  |  |
| No | 202 | 54 (43.9) | 148 (42.8) |  |  |  |  |
| **Sex** |  |  |  | 0.345 |  |  | 0.196 |
| Male | 269 | 75 (61.0) | 194 (56.1) |  | 160 (59.9) | 109 (54.0) |  |
| Female | 200 | 48 (39.0) | 152 (43.9) |  | 107 (40.1) | 93 (46.0) |  |
| **Age (year)** |  |  |  | 0.050 |  |  | 0.412 |
| ≤ 60 | 239 | 55 (23.0) | 184 (77.0) |  | 141 (59.0) | 98 (41.0) |  |
| 60-80 | 200 | 55 (27.5) | 145 (72.5) |  | 112 (56.0) | 88 (44.0) |  |
| > 80 | 30 | 13 (43.3) | 17 (56.7) |  | 14 (46.7) | 16 (53.3) |  |
| **Grade** | |  |  | 0.280 |  |  | 0.125 |
| Well | 42 | 11 (8.9) | 31 (9.0) |  | 18 (6.7) | 24 (11.9) |  |
| Moderate | 329 | 80 (65.0) | 249 (72.0) |  | 189 (70.8) | 140 (69.3) |  |
| Poor | 98 | 32 (26.0) | 66 (19.1) |  | 60 (22.5) | 38 (18.8) |  |
| **Subsite** |  |  |  | 0.101 |  |  | 0.225 |
| Tongue | 286 | 84 (68.3) | 202 (58.4) |  | 160 (59.9) | 126 (62.4) |  |
| Gum | 31 | 11 (8.9) | 20 (5.8) |  | 13 (4.9) | 18 (8.9) |  |
| Floor of mouth | 75 | 13 (10.6) | 62 (17.9) |  | 45 (16.9) | 30 (14.9) |  |
| Palate | 17 | 3 (2.4) | 14 (4.0) |  | 9 (3.4) | 8 (4.0) |  |
| Others | 60 | 12 (9.8) | 48 (13.9) |  | 40 (15.0) | 20 (9.9) |  |
| **T stage** | |  |  | 0.919 |  |  | 0.138 |
| T1 | 223 | 58 (47.2) | 165 (47.7) |  | 119 (44.6) | 104 (51.5) |  |
| T2 | 246 | 65 (52.8) | 181 (52.3) |  | 148 (55.4) | 98 (48.5) |  |
| **Race** |  |  |  | 0.571 |  |  | 0.893 |
| White | 413 | 107 (25.9) | 306 (74.1) |  | 236 (57.1) | 177 (42.9) |  |
| Black | 23 | 5 (21.7) | 18 (78.3) |  | 12 (52.2) | 11 (47.8) |  |
| Other | 33 | 11 (33.3) | 22 (66.7) |  | 19 (57.6) | 14 (42.4) |  |
| **Marital status** |  |  |  | 0.224 |  |  | **0.022** |
| Married | 266 | 76 (28.6) | 190 (71.4) |  | 166 (62.4) | 100 (37.6) |  |
| Unmarried | 186 | 45 (24.2) | 141 (75.8) |  | 92 (49.5) | 94 (50.5) |  |
| Unknown | 17 | 2 (11.8) | 15 (88.2) |  | 9 (52.9) | 8 (47.1) |  |

LNE, Lymph nodes examined; PORT, postoperative radiotherapy.
